# Supplementary material for: Improving Knowledge of Audit and Feedback among Health Care Professionals in Sicily
Source: Healthcare (Basel). 2023 Jul 9;11(14):1987. doi: 10.3390/healthcare11141987 (PMC10378836; doi:10.3390/healthcare11141987)
Supplement: Supplementary file 1 [file healthcare-11-01987-s001.zip › healthcare-2468405-supplementary.pdf]

# 1 Supplementary Figures and Tables

## 1.1 Supplementary Figures

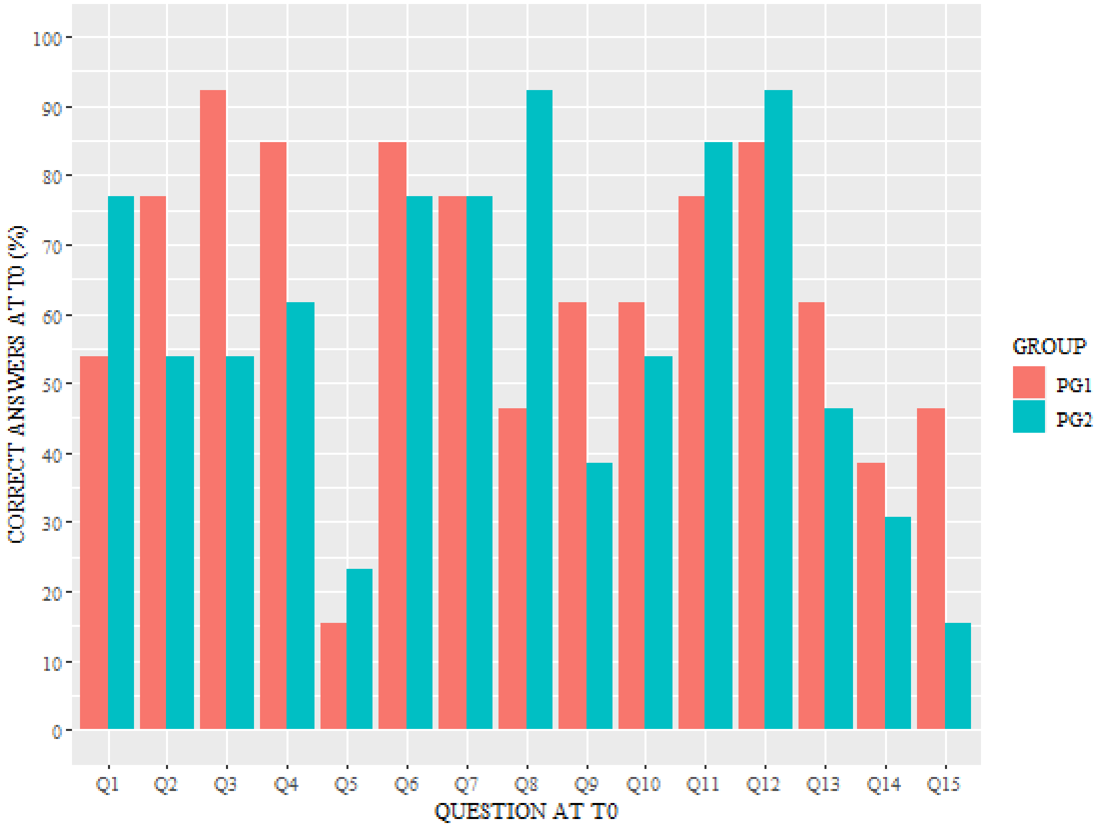


**Supplementary Figure S1.** Percentage of correct answers per participant group at T0.
Legend: Q1-15 = Question 1-15; PG1-2 = Participant Group 1-2.

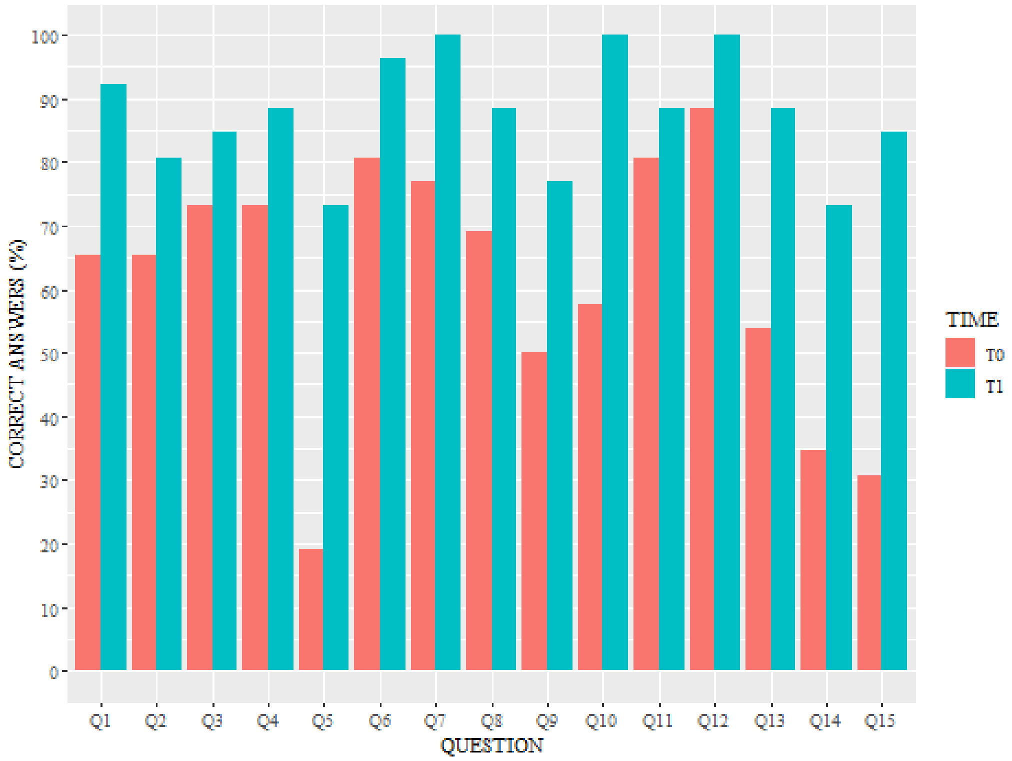


**Supplementary Figure S2.** Percentage of correct answers for all questions at T0 and T1.
Legend: Q1-15 = Question 1-15.

## 1.2 Supplementary Tables

| QUESTION | PG1 | | PG2 | | ALL | |
|---|---|---|---|---|---|---|
| | T0 | T1 | T0 | T1 | T0 | T1 |
| Q1 | 7 | 11 | 10 | 13 | 17 | 24 |
| Q2 | 10 | 11 | 7 | 10 | 17 | 21 |
| Q3 | 12 | 12 | 7 | 10 | 19 | 22 |
| Q4 | 11 | 13 | 8 | 10 | 19 | 23 |
| Q5 | 2 | 9 | 3 | 10 | 5 | 19 |
| Q6 | 11 | 13 | 10 | 12 | 21 | 25 |
| Q7 | 10 | 13 | 10 | 13 | 20 | 26 |
| Q8 | 6 | 11 | 12 | 12 | 18 | 23 |
| Q9 | 8 | 11 | 5 | 9 | 13 | 20 |
| Q10 | 8 | 13 | 7 | 13 | 15 | 26 |
| Q11 | 10 | 12 | 11 | 11 | 21 | 23 |
| Q12 | 11 | 13 | 12 | 13 | 23 | 26 |
| Q13 | 8 | 12 | 6 | 11 | 14 | 23 |
| Q14 | 5 | 9 | 4 | 10 | 9 | 19 |
| Q15 | 6 | 12 | 2 | 10 | 8 | 22 |

**Supplementary Table S1.** Number of correct answers for all questions at T0 and T1 per participant group.
Legend: Q1-15 = Question 1-15; PG1-2 = Participant Group 1-2.
